# Pride, Love, and Twitter Rants: Combining Machine Learning and Qualitative Techniques to Understand What Our Tweets Reveal about Race in the US

**DOI:** 10.3390/ijerph16101766

**Published:** 2019-05-18

**Authors:** Thu T. Nguyen, Shaniece Criss, Amani M. Allen, M. Maria Glymour, Lynn Phan, Ryan Trevino, Shrikha Dasari, Quynh C. Nguyen

**Affiliations:** 1 Department of Epidemiology & Biostatistics, University of California San Francisco, San Francisco, CA 94158, USA; mglymour@gmail.com (M.M.G.); shrikhadasari26@gmail.com (S.D.); 2 Department of Health Science, Furman University, Greenville, SC 29613, USA; shaniece.criss@furman.edu; 3 Divisions of Community Health Sciences and Epidemiology, University of California, Berkeley, CA 94704, USA; amaniallen@berkeley.edu; 4 Department of Social and Behavioral Sciences, Harvard T.H. Chan School of Public Health, Boston, MA 02215, USA; 5 Program of Public Health Science, University of Maryland School of Public Health, College Park, MD 20742, USA; lkphan@outlook.com; 6 Department of Health Sciences, College of Science and Health, DePaul University, Chicago, IL 60614, USA; rtrevin3@mail.depaul.edu; 7 Department of Epidemiology & Biostatistics, University of Maryland School of Public Health, College Park, MD 20742, USA; qtnguyen@umd.edu

**Keywords:** social media, minority groups, discrimination, big data, content analysis

## Abstract

*Objective*: Describe variation in sentiment of tweets using race-related terms and identify themes characterizing the social climate related to race. *Methods*: We applied a Stochastic Gradient Descent Classifier to conduct sentiment analysis of 1,249,653 US tweets using race-related terms from 2015–2016. To evaluate accuracy, manual labels were compared against computer labels for a random subset of 6600 tweets. We conducted qualitative content analysis on a random sample of 2100 tweets. *Results*: Agreement between computer labels and manual labels was 74%. Tweets referencing Middle Eastern groups (12.5%) or Blacks (13.8%) had the lowest positive sentiment compared to tweets referencing Asians (17.7%) and Hispanics (17.5%). Qualitative content analysis revealed most tweets were represented by the categories: negative sentiment (45%), positive sentiment such as pride in culture (25%), and navigating relationships (15%). While all tweets use one or more race-related terms, negative sentiment tweets which were not derogatory or whose central topic was not about race were common. *Conclusion*: This study harnesses relatively untapped social media data to develop a novel area-level measure of social context (sentiment scores) and highlights some of the challenges in doing this work. New approaches to measuring the social environment may enhance research on social context and health.

## 1. Introduction

A social climate in which greater hostility towards minorities is manifested may cause psychological stress and increase risk of negative health outcomes. 

Experiences with discrimination are commonly measured at the individual level by self-report [1,2]. Self-reported racial attitudes and beliefs are subject to a number of limitations including social desirability bias and self-censorship [3,4], risking invalid exposure assessment [5,6]. Self-reports of racial discrimination are also subject to reporting bias. This may include, among other things, coping (e.g., denial), trait- or state-based aspects of personality (e.g., stigma consciousness, race-based rejection sensitivity), and aspects of racial identity (e.g., internalized racism) [5]. Cognitive measures have been developed to assess implicit racial bias [7,8]. Audit studies have also documented discrimination in a variety of areas including housing [9] and employment [10]. However, all these measures are aimed at characterizing individual level experiences and bias.

While individual level measures of racial bias and discrimination are valuable in documenting individual experiences, the social climate of a place can provide an ecological perspective for understanding one’s experiences in relation to the broader social environment. An ecosocial approach to the study of discrimination views discrimination as operating across multiple levels over the life course and reflecting systemic prejudice, which has emergent properties of its own despite individual level experiences. For example, a landmark study examined the influence of contextual indicators of discrimination on birth outcomes among pregnant women of Arab descent. Comparing birth outcomes for women by race/ethnicity and nativity for the 6 months after 11 September 2001, to the same six-month period one year prior, only Arabic-named women experienced significantly increased risk of preterm birth and low birth weight post September 2001 [11]. The study did not measure individual women’s personal exposure to harassment, discrimination, violence, etc. However, after 11 September 2001, the social climate for Arabic-named women had changed, and was associated with increased risk of adverse birth outcomes for this population. Prejudice, antipathy towards a group based on poorly founded generalizations [12], has broad and important implications for health and development. This, and other studies, provides evidence relating racial prejudice to the health of communities [13]. 

Social media data offer an increasingly popular data resource for assessing social climate. Social media provides a space and opportunity for people to publicly express their ideas and viewpoints and represents what many believe to be a relatively untapped resource for assessing the contextual level social climate related to race. With 21% of US adults using Twitter and over 90% of Twitter users making their profile and communication public [14], social media provides researchers the opportunity to examine the public communications of a substantial proportion of the country. Social media therefore presents some advantages in illuminating national and potentially place-specific sentiments about race/ethnicity, providing a “temperature” of the social environment where the tweets are written. On Twitter, users are not required to report their age, sex, race, or geographic location, and as a result, grants people a level of anonymity. Studies have found that people feel less inhibited in expressing their views and beliefs online compared to in-person interactions [15,16,17]. Twitter and other social media data have been used to describe national patterns in happiness, diet, and physical activity [18,19]; examine beliefs, attitudes, and sentiment towards various topics (e.g., vaccinations) [20]; track health behaviors and perform health surveillance [21], and investigate patient-perceived quality of care [22].

Few studies have used social media to examine more sensitive topics such as race and racism online. A previous study on internet-based racism found area-level racism, operationalized as the proportion of Google searches containing “n-word” was positively associated with all-cause Black mortality [23]. Like this study, prior studies have primarily focused on the use of racial slurs [24,25]. However, terms conventionally perceived as racial slurs can be used in non-derogatory ways, and such re-appropriation is common on Twitter; for instance, in popular culture the term “n*gga” is often used as an in-group term without valuation [24], making assessment of racial sentiment challenging. Furthermore, discussions conveying racial sentiment can occur without the use of racial slurs. A more comprehensive examination of tweets using race-related terms may include a sentiment analysis of tweets using racial slurs as well as neutral racial terms such as “Black”, “African American”, or “Asian”. Fewer positive tweets using race-related terms in a state or county may indicate an environment that is less welcoming to racial and/or ethnic minorities, which may be a source of stress. A greater number of positive tweets using race-related terms may be indicative of an environment that embraces racial and ethnic diversity and is more inclusive. Therefore, examining positive and non-positive tweets may provide a fuller picture of the social context of a place.

To provide a measure of social climate in relation to race and ethnicity and address prior limitations of self-reported, individual-level measures, we employed mixed methods to (1) examine variation in sentiment regarding tweets using one or more race-related terms, and (2) identify emerging themes of the tweets. This paper describes the collection and sentiment analysis of Twitter data using one or more race-related terms and the qualitative content analysis of a subsample of tweets to provide a more contextual-level understanding of the social climate related to race.

## 2. Methods

### 2.1. Social Media Data Collection and Processing

From March 2015–April 2016, we utilized Twitter’s Streaming Application Programming Interface (API) to continuously collect a random sample of publicly available tweets. Twitter’s Streaming API gives users access to a random 1% sample of tweets. The Twitter API is freely available to everyone and this API allows users free access to subsets of public tweets. Users may request tweets for a certain geographic area that contain a particular set of keywords, or just random subsets of tweets. Depending on the search criteria used by the researcher, the number of tweets returned may comprise less than 1% of all available tweets. In our case, we restricted the data collection to tweets with latitude and longitude coordinates that were sent from the contiguous United States (including District of Columbia). We dropped duplicate tweets according to their “tweet_id” (each tweet has a unique identification). We removed job postings according to the hashtags “#job” and “#hiring.” We manually examined outliers in our datasets (the top 99th percentile of tweeters) and eliminated automated accounts and accounts for which the majority of tweets were advertisements. In total, we collected 79,848,992 million general topic tweets from 603,363 unique Twitter users.

To identify potentially race-related tweets, a keyword list of 398 race-related terms was compiled (online supplementary materials Table S1 with the most commonly occurring terms bolded) from racial and ethnic categories used by the US census and an online database of racial slurs [26]. Tweets using at least one or more of the race-related terms were identified resulting in a final analytic dataset of 1.25 million tweets. Location information from the tweets was used to map the tweets to their respective county and state using Python and R-tree to build the spatial index [27,28]. Count of tweets using race-related terms by state can be found in the online supplementary materials (Table S2). This study was determined exempt by the University of California, San Francisco Institutional Review Board (Ref: 18-24255).

### 2.2. Computer Modeling: Sentiment Analysis of Twitter Data

To prepare the dataset of tweets for analysis by the computer algorithm, each tweet was divided into tokens, which roughly correspond to words, using the Stanford Tokenizer [29], an open access software tool. Below, we briefly describe the algorithms we used to create variables for sentiment.

To conduct sentiment analysis on tweets with references to racial and ethnic minorities, we used the Stochastic Gradient Descent Classifier (SGD), an optimization method that minimizes a given loss function [30], in Python software version 2.7 (Python Software Foundation, Wilmington, DE, USA). In SGD, weights in the sentiment models are updated for each example in the training dataset [30], and several iterations are made over the training data until the algorithm converges. A strength of SGD is that it is quick to train and has been applied to large-scale and sparse-learning problems [31]. Preprocessing of the tweets was undertaken to remove inconsequential variation and allow the sentiment model to focus on the relevant features of the tweets [32]. These preprocessing steps included removing stop words (e.g., the, a, is), additional white space, punctuations, hashtag symbols, URLs, and Twitter usernames. All words were converted to lowercase, and the repetition of a character (symbol or alphabet letter) was replaced by one instance of the character.

Labeled tweets from Sanders Analytics (*n* = 5513 tweets) [33] and Kaggle (*n* = 7086 tweets) [34], and emoticons derived from Sentiment140 (e.g., smiley face to indicate happiness, *n* = 1.6 million tweets) [35] were used to train the computer algorithm to analyze the tweets. The computer algorithm then uses these labeled tweets to learn what a human considers a “positive” or “negative/neutral” tweet. Once trained, the computer algorithm then categorizes “positive” and “negative/neutral” tweets from our sample of 1.25 million tweets.

Preliminary analyses revealed that the SGD algorithm’s accuracy against manual annotations was substantially greater for dichotomous sentiment compared to multiple sentiment categories. Specifically, the model was able to distinguish positive vs. non-positive tweets but had much lower accuracy when three (positive, neutral, and negative) categories were used. According to the race-related terms referenced in the tweet, we grouped tweets into four main racial/ethnic categories: Blacks, Hispanics, Asians, and Middle Eastern. The latter included tweets that were anti-Islamic or related to Muslims.

Tweets were assigned values of 1 (for positive) or 0 (for negative/neutral). State-level sentiment variables were created by averaging the dichotomous sentiment of tweets referencing various racial/ethnic groups. Next, state-level sentiment was mapped in order to examine geographic variation. Statistical analyses were implemented with Stata MP15 (StataCorp LP, College Station, TX, USA).

After training the computer algorithm and conducting the sentiment analysis on our novel Twitter dataset, we evaluated the accuracy of the computer algorithm using a randomly selected, manually labeled subset of tweets. Three coauthors (LP, TR, and SD) categorized the sentiment of the tweet as negative, neutral, or positive. In addition to labeling for sentiment, the coders indicated whether the tweet used discriminatory or stereotyping language about a racial or ethnic group (Yes/No). A sample of 150 tweets was labeled by all coders, and discrepancies were resolved. Coders then analyzed 150 more tweets, and an acceptable level of agreement was reached (kappa = 90%). Coders then independently coded 2000 tweets. Agreement on the coding of positive and non-positive tweets between the computer algorithm and the manual labels was computed. Please see Figure S1 in the online supplementary materials for a diagram presenting the analytic sample and analysis steps.

### 2.3. Content Analysis

A content analysis exploring themes emerging from a random sample of 2100 tweets using race-related terms was conducted. Two members of the research team developed a codebook (i.e., a list of codes and definitions representing the emerging themes) based on a literature review and coding and discussing 150 tweets from the above sample. This consensus building process enhanced the codebook by clarifying operational definitions. The final categories of tweets included an overall categorization of sentiment (negative, positive, or neutral) and the following sub-headings by sentiment conveying the emergent themes: casual use/slang, food, stereotypes, and related to sexuality/relationships. Complaint, insult, and hostility were additional sub-headings specific to negative sentiment. Using this coding scheme, two members of the research team coded a randomly selected sample of 2100 tweets. The researchers double-coded 300 tweets throughout the process (100 tweets in the beginning, 100 tweets in the middle, and 100 tweets near the end of the sample). Any disagreements in coding were discussed until consensus was met. Kappa agreement of 80% was met for each inter-coder session. In addition to the 300 double-coded tweets, each of the two team members independently coded 900 tweets.

## 3. Results

Of the 6600 tweets using a racial term which were manually coded, about 25% were coded as positive, 44% as neutral, and approximately 30% as negative. Only a small proportion (about 6%) of tweets with a race-related word used discriminatory or stereotyping language. Agreement on the dichotomous characterization between computer labels and manually generated labels was 74%, which is similar to the 76% accuracy found in a prior study labeling racist tweets [36].

### 3.1. Quantitative Analyses: Descriptive Characteristics of Tweets

Below, we describe results from our quantitative analyses, based on the SGD algorithmic classification of the sentiment (positive vs. neutral/negative) of 1,249,653 tweets containing at least one of the relevant keywords pertaining to a racial or ethnic minority group. Descriptive details of these tweets have been previously described [37]. Briefly, approximately 620,000 tweets were about Blacks, 205,000 about Hispanics, 270,000 about Asians, and 60,000 about Middle Eastern groups. From a list of 398 terms, only 20 terms were necessary to characterize 84% of all tweets with references to a racial or ethnic minority group (top 20 terms bolded in list in Appendix A Table A1).

The top Twitter terms were “n*gga” (42.6%) (please note that when “*” is present, it was inserted by the study team and not part of the original text), “Mexican” (8.4%), “Thai” (4.2%), and “Asian” (4.0%). The automated sentiment classification characterized the tweet overall. Most tweets including a racial term were not specifically about race. We found that a “positive” tweet does not necessarily imply a positive racial sentiment, but merely a positive sentiment in which a racial term was used. For example, “@username I love you, n*gger.” Overall, 15.2% of tweets using race-related terms expressed a positive sentiment; 13.1% of tweets containing the word “n*gga” were positive. The term “n*gger” was exceedingly rare, with only 507 mentions in our entire dataset, of which 12.7% were positive in sentiment.

**Figure 1:**
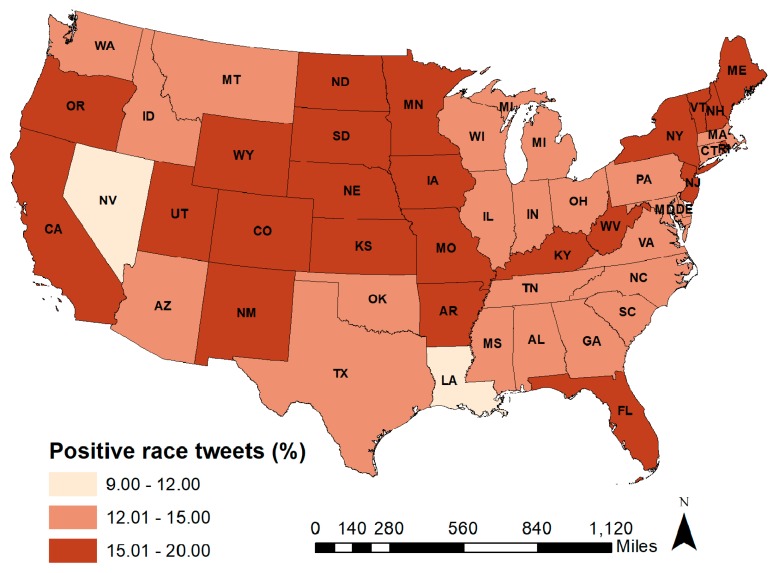
Geographic distribution of percent tweets using race-related terms that are positive, collected April 2015–March 2016.

**Figure 2:**
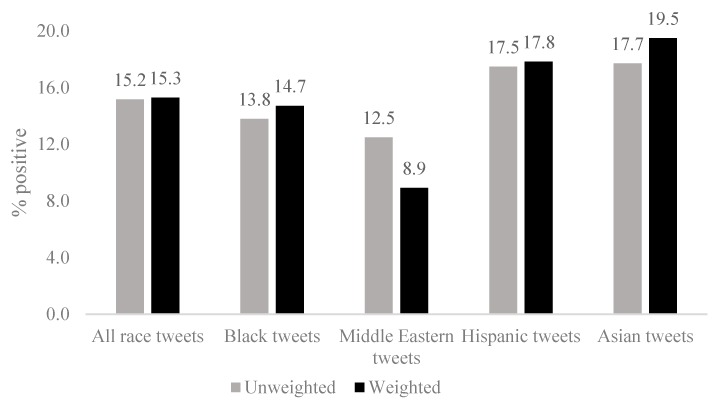
Descriptive characteristics for sentiment of 2015–2016 tweets using race-related terms. Data sources: 1,249,653 geolocated tweets from the 48 contiguous United States plus the District of Columbia collected between April 2015–March 2016. Number of followers was used to calculate weighted percentages.

States where tweets using a race-related term were least likely to express a positive sentiment were Nevada and Louisiana with 9–12% of tweets being positive. States with the most positive sentiment tweets were Utah, Oregon, and North Dakota with 17–20% of tweets being positive (Figure 1). Across the United States, tweets referencing Asians (17.7%) and Hispanics (17.5%) had the highest percentage of positive sentiment. Tweets that referenced Middle Eastern groups had the lowest positive sentiment (12.5% positive) followed by tweets that reference Blacks (13.8% positive). (See online supplementary materials Figures S2–S5 for maps of positive sentiment by race/ethnicity). When we weighted tweets by the number of followers (i.e., tweets sent by a user with more followers were weighted more heavily than tweets sent by a user with fewer followers), positive sentiment increased at least slightly for tweets using Black, Hispanic, and Asian related terms, but not for tweets using a term related to Middle Eastern groups. For Middle Eastern groups, the weighted percentage of positive tweets, 8.9%, was lower than the unweighted percentage of 12.5% (see Figure 2). We examined variation in sentiment by race across months and found relatively stable sentiment that maximally differed by 3–5% (Table 1).

### 3.2. Qualitative Content Analysis: Themes

In the random sample of 2100 tweets for the thematic analysis, 45% had themes expressing a negative sentiment, 25% had themes expressing a positive sentiment, and 15% had themes connected to intimate relationships (including negative and positive sentiment). Two percent of tweets were derogatory or expressed a racial/ethnic stereotype (Table 2). While all tweets use one or more race-related terms, the central topic for many of the tweets was not about race.

#### 3.2.1. Spectrum of Negativity

Tweets ranged from innocuous statements about daily life, to complaints, insults using general derogatory language, and expressed hostility mentioning violence. Although uncommon, some tweets also mentioned racial or ethnic stereotypes or were racially derogatory (e.g., “Middle Eastern/Arabic accents piss me off more than most things,” Table 2). The use of “n*gga” was pervasive in the negative sentiment tweets. However, most of the tweets using this term were not derogatory. Rather, Twitter users most frequently use this term casually as slang. The use of profanity was common in negative tweets.

#### 3.2.2. Pride

Most of the positive sentiment tweets related to pride. Some Twitter users indicated love for their culture and food. Some tweets had a tone of pride for being loyal to their friends throughout the years. In addition, several tweets were rejecting negative views of their race and offering alternative viewpoints.

#### 3.2.3. Intimate Relationships

Many of the tweets focused on some aspect of an intimate relationship. Specifically, these tweets included comments about appearance, affinity to a particular race, cheating, frustration over behavior of men or women, sex, and introspection about one’s own behavior. “N*gga” was a common term used in the relationship tweets, though it was not generally used as a racial slur but rather as slang. Many tweets about appearance and affinity towards a particular race regarding dating and marriage were positive. The tweets about cheating, sex, and frustration over the behavior of men or women were mainly negative and many times included profanity, but were not generally about race although a race-related term was used. On the other hand, racial terms were, at times, used to aid in an insult, such as tweets about cheating and unfavorable views about men or women (“N*ggas only lie cus females can’t handle the 100% truth”, Table 2). Some users’ tweets indicated introspection and reflection about past and future relationships (I’m f*cking up by pushing people away and acting like a n*gga,” Table 2).

## 4. Discussion

In this study, we examined tweets using a race-related term that were collected over a year-long period. Sentiment analysis on the full sample of 1.25 million tweets using natural language processing revealed sentiment expressed in tweets differed across the race/ethnic group mentioned. Tweets mentioning Middle Eastern groups or Blacks were least likely to express positive sentiments. Sentiment varied moderately across calendar month, with the maximum variation being about 2–5%, indicating sentiment at the state level was relatively stable over the one-year period. The quantitative analysis revealed that tweets that expressed discriminatory sentiments were rare, highlighting the need for a more in-depth analysis of tweets utilizing race-related terms. Therefore, we conducted a qualitative content analysis of a random sample of 2100 tweets that elucidated three core themes: spectrum of negativity, pride, and various aspects of intimate relationships. Understanding the nuances of the sentiment can provide more insight about whether and why patterns in tweet sentiments could plausibly be linked to a particular health outcome.

Tweets may not represent beliefs. Tweets may reflect an image a person may be promoting or another motive. However, because tweets are expressed statements that people are willing to make publicly, they may reflect an area’s social acceptability or willingness to showcase certain sentiments. As such, we leverage tweets to gauge the racial climate of a state. State-level differences in Twitter activity can reflect changes in willingness to vocalize negative sentiment. Increased vocalization could be an additional source of stress. Whereas before, negative sentiment was more hidden, changes in public acceptance or willingness to display negative sentiment can be an additional source of stress and impact health and well-being. Previous studies have found that social media can capture information about the social environment that has utility for predicting health outcomes. For example, Eichstaedt et al. found greater use of anger, negative emotion, and disengagement words on Twitter predicted county-level heart disease mortality [38]. Notably, previous research found more negative area-level sentiment towards blacks and Middle Eastern groups was related to worse individual-level birth outcomes, and this was true for the full population and for racial minorities [37]. Thus, a hostile social climate related to race may have implications for health and well-being, but developing valid and inexpensive measures remains an important research challenge.

In this paper, social media data were used to examine sentiment towards racial/ethnic minorities. Limitations of the computer algorithm include the inability to identify sarcasm or humor. In addition, the tweets using one or more race related terms may be a part of a conversation but only tweets using specific-race related keywords were analyzed. Thus, the context in which the tweet is embedded and the context in which the specific racial keyword is used may be lost.

The content analysis revealed the central topic of numerous tweets, despite using one or more race-related terms was not, in fact, about race. Our analysis was conducted at the state level, and there may be individuals within a smaller area that are exposed to a higher proportion of derogatory tweets than the state average. Overall, negative sentiment tweets that were not racially derogatory or making stereotypical statements were common. For example, one of the most frequently used race-related term was n*gga. While many tweets using this term had a negative sentiment in terms of the context of the tweet, the term itself was often used casually as slang. The content analysis revealed more clearly the limitations of the sentiment analysis to identify tweets that were not centrally related to race although using race-related terms. Nonetheless, the tweets using at least one of the 398 race-related terms covering a range of topics may represent a signal of the average level of racial attitudes, given our prior work showing an association between state-level sentiment scores and preterm, low birth weight, and very low birth weight [37]. Future work can develop algorithms to categorize tweets by sentiment as well as by topic.

The present analysis was based solely on the text within the tweet; images and videos could not be analyzed. We used race-related keywords to identify tweets. However, tweets that do not use racial slurs or neutral racial terms may be race-related. Previous studies have found 1–2% of all tweets have latitude and longitude coordinates [39]. The analysis includes only geotagged tweets, which may vary from tweets without geotagged information. This study assessed the social environment via online tweets, and these may differ from in-person interactions. However, when assessing expressed attitudes and beliefs online, people may feel less inhibited due to anonymity and invisibility of being online [15].

In the current study, tweets sent from that state were utilized to construct indicators of sentiment towards racial/ethnic minorities. Social media users tend to be younger than the general population with 36% of people 18–29 using Twitter in 2016 while 10% among those 65+ years reporting Twitter use [40]. Thus, Twitter users may not be representative of the broader US population. However, social media has been increasing over time, and access to the internet using cellphones has resulted in people from all socioeconomic backgrounds using Twitter. Tweets also include information seldom found in conventional data sources with researchers being able to capture real-time conversations about daily life, activities, and beliefs.

## 5. Conclusions

This paper is among the first to characterize the sentiment of tweets using race-related terms. In this paper, we address the limitations of self-reported measures of discrimination and racial bias to create an environmental indicator of the social context (state-level sentiment scores) from expansive and relatively untapped social media data. Self-reported measures provide valuable information about individual-level experiences, but these measures may be subject to reporting bias. Online discussions using race-related terms can be used to better understand the social context for areas across the United States. However, this area of research is in its infancy. Importantly, our findings revealed some of the challenges and complexities of characterizing tweets using race-related terms and the importance of better understanding what, exactly, social media data are capturing. The study findings highlight the difference between sentiment of the tweets, the topic of the tweet, and the ways in which the racial terms are being used (e.g., in derogatory or neutral ways). Despite these challenges, there remains much to be learned from these data. Future work is needed to further identify and categorize race-specific tweets, and to examine whether the social context influences the health, economic, social, and educational outcomes of minorities and immigrants to the United States and thus identify levers for alleviating disparities.

## Figures and Tables

**Table 1:** Twitter-derived sentiment by month of year, 2015–2016.

Month	Percent of Tweets That Are Positive				
	All Race	Blacks	Middle Eastern	Hispanic	Asian
Jan	15.7%	15.6%	11.4%	17.0%	16.2%
Feb	18.1%	16.2%	12.4%	17.8%	20.9%
Mar	16.0%	15.0%	11.5%	18.0%	16.4%
Apr	14.7%	13.3%	13.4%	18.0%	19.3%
May	15.5%	13.7%	14.0%	17.8%	18.3%
Jun	15.4%	13.9%	12.4%	16.5%	18.3%
Jul	15.0%	14.1%	11.5%	15.1%	17.2%
Aug	14.7%	14.1%	11.8%	14.8%	15.8%
Sep	15.0%	13.5%	10.8%	15.6%	17.6%
Oct	15.6%	14.7%	11.0%	16.6%	16.8%
Nov	15.2%	15.6%	9.6%	15.7%	16.7%
Dec	14.9%	14.7%	9.7%	16.1%	16.0%
Maximum difference	3.4%	2.8%	4.4%	3.2%	5.1%

Data sources: 1,249,653 geolocated tweets from the 49 contiguous United States collected between April 2015–March 2016.

**Table 2:** Content analysis themes of 2015–2016 US tweets with illustrative Examples, 2015–2016.

Themes	Example Tweets
**Negative Sentiment**	
Innocuous	▪ Can’t Watch The (professional basketball team) Play. These N*ggas Boring AF
Complaints	▪ N*ggas don’t bring sh*t but headaches
Insults using derogatory language	▪ You bend over backwards for that n*gga you a f*ggot ▪ Some girls really need to dye their fkn hair and maintain that weave looking good #sh*tsghetto ▪ Stupid a*s ho*s and n*ggas bruh
Generalizations, use of racial slurs in derogatory ways	▪ I work like a freaking Mexican ▪ I seriously hate when I hear about Desi or Arab mothers preferring lighter skinned brides for their sons. Stop this stupid mentality. ▪ Middle Eastern/Arabic accents piss me off more than most things. ▪ You can’t use big words around hood n*ggas ▪ That gook got so mad he was steamed rice
Hostile tweets, some mentioning violence	▪ N*gga gon break his Kneecaps [url] ▪ And if they are carrying a Mexican flag in Az. they need to be arrested.
**Positive Sentiment**	
Cultural pride	▪ asian at heart, forever ▪ mexican anything is the best way to my heart tbh ▪ #AskFluffy Why do you love being Mexican?
Food	▪ God bless Mexicans and their delicious food <33,343 [url] ▪ Mexican food and family can definitely turn a day around ▪ We are getting Chinese food for lunch. I repeat. We are getting Chinese food for lunch.
Loyalty within friendships	▪ Stil down with my day one n*ggas! ▪ Don’t worry bout my n*ggas cuz I gottem ▪ I’ll never leave my n*ggas hanging
Denying stereotypes	▪ I don’t think I’m ghetto at all..... ▪ N*ggas that are locked up are prolly some of the smartest most innovative people on earth ▪ Everybody wanna be Haitian now wasn’t like that bout 15 years ago Lol
**Intimate Relationships**	
Appearance	▪ damn n*gga you sexy af ▪ yall cute little n*gga
Affinity to a particular race	▪ Middle eastern men are so fine ▪ I’m going to marry a Cuban or Columbian, that’s final ▪ Nothing will make me happier than a Hispanic woman I love Jesus but I like Jewish boys”―sh*t Shannon says ▪ I love black men.....
Cheating	▪ F*ck unfaithful n*gga ▪ I’m in love with a n*gga that got endless b*tches
Frustration over behavior of men or women	▪ If you trippin over a n*gga, you a weak ho*. Vice versa ▪ N*ggas only lie cus females can’t handle the 100% truth ▪ Females hurt themselves knowing the same n*gga will hurt them
Sex	▪ Some girls think that stuff being given to you is a pass of generosity, when n*ggas want to just catch that p*ssy.
Introspection about own behavior	▪ I’m f*cking up by pushing people away and acting like a n*gga ▪ Im not in the business of keep a n*gga that aint tryna be kept

When “*” is present, it was inserted by the study team and not part of the original text. Data sources: 1,249,653 geolocated tweets from the 49 contiguous United States collected between April 2015–March 2016.

## Supplementary Materials

The following are available online at https://www.mdpi.com/1660-4601/16/10/1766/s1, Figure S1: Analytic Sample and Analysis Flow Chart, Figure S2: Geographic distribution of percent tweets using Black-related terms that are positive, collected April 2015–March 2016, Figure S3: Geographic distribution of percent tweets using Middle Eastern-related terms that are positive, collected April 2015–March 2016, Figure S4: Geographic distribution of percent tweets using Hispanic-related terms that are positive, collected April 2015–March 2016, Figure S5: Geographic distribution of percent tweets using Asian-related terms that are positive, collected April 2015–March 2016, Table S1: Race terms used in Twitter data Collection, Table S2: Count of tweets using race-related terms by state.

## Appendix A

**Table A1:** Race terms used in Twitter data Collection with most commonly occurring terms bolded.

Items	Race
afghanistan	Middle Eastern
afghanistani	Middle Eastern
afghans	Middle Eastern
african american	Black
african americans	Black
african’t	Black
africoon	Black
afro caribbean	Black
afro-caribbean	Black
aid refugees	refugees
alaska native	Alaskan Native
american indian	Native American
apache indian	Native American
apache nation	Native American
apache tribe	Native American
arab	Middle Eastern
arabs	Middle Eastern
arabic	Middle Eastern
arabush	Arab
**asian**	Asian
asians	Asian
asian indian	Asian
bahamian	Black
bahamians	Black
bamboo coon	Asian
ban islam	anti-islamic
ban muslim	anti-islamic
ban on mulsims	anti-islamic
bangalees	Asian
bangladeshi	Asian
banislam	anti-islamic
banjo lip	Black
banmuslim	anti-islamic
banonmulsims	anti-islamic
bantu	Black
beaner	Mexican
beaner shnitzel	Multi-race
beanershnitzel	Multi-race
bengalis	Asian
bhutanese	Asian
biscuit lip	Black
bix nood	Black
black boy	Black
black boys	Black
black female	Black
black girl	Black
black girls	Black
black male	Black
black men	Black
black women	Black
blacks	Black
bootlip	Black
borde jumper	Hispanic
border bandit	Hispanic
border control	immigrant
border fence	immigrant
border hopper	Hispanic
border nigger	Hispanic
border security	immigrant
border surveillance	immigrant
border wall	immigrant
bow bender	Native American
brazilians	Hispanic
buffalo jockey	Native American
build a wall	immigrant
buildawall	immigrant
bumper lip	Black
burmese	Asian
burnt cracker	Black
burundi	Black
bush-boogie	Black
bushnigger	Native American
cairo coon	Middle Eastern
cambodian	Asian
cambodians	Asian
camel cowboy	Middle Eastern
camel fucker	Middle Eastern
camel jacker	Middle Eastern
camelfucker	Middle Eastern
camel-fucker	Middle Eastern
cameljacker	Middle Eastern
camel-jacker	Middle Eastern
carpet pilot	Middle Eastern
carpetpilot	anti-islamic
carribean people	Black
caublasian	Multi-race
central american	Hispanic
chain dragger	Black
chamorro	Asian
cherokee indian	Native American
cherokee nation	Native American
cherokee tribe	Native American
cherry nigger	Native American
chexican	Multi-race
chicano	Hispanic
chicanos	Hispanic
chiegro	Asian
chinaman	Asian
**chinese**	Asian
ching-chong	Asian
chink	Asian
chinks	Asian
chippewa indian	Native American
chippewa nation	Native American
chippewa tribe	Native American
choctaw indian	Native American
choctaw nation	Native American
choctaw tribe	Native American
clit chopper	Middle Eastern
clit-chopper	Middle Eastern
clitless	Middle Eastern
clit-swiper	Middle Eastern
coconut nigger	Asian
colombian	Hispanic
columbians	Hispanic
congo lip	Black
congolese	Black
coonass	Black
coon-ass	Black
coontang	Black
costa rican	Hispanic
cracker jap	Asian
**cuban**	Hispanic
cubans	Hispanic
dampback	Hispanic
darkey	Black
darkie	Black
darky	Black
deport	immigrant
deportation	immigrant
deported	immigrant
deporting	immigrant
deports	immigrant
derka derka	anti-islamic
derkaderka	anti-islamic
diaper head	Middle Eastern
diaperhead	Middle Eastern
diaper-head	Middle Eastern
dog muncher	Asian
dog-muncher	Asian
**dominican**	Hispanic
dominicans	Hispanic
dothead	South Asians
dune coon	Middle Eastern
dune nigger	anti-islamic
dunecoon	anti-islamic
dunenigger	anti-islamic
durka durka	Middle Eastern
durka-durka	Middle Eastern
east asian	Asian
ecuadorian	Hispanic
egyptian	Black
egyptians	Black
end sanctuary	immigrant
ethiopian	Black
ethiopians	Black
fence fairy	Hispanic
fence hopper	Hispanic
fence-hopper	Hispanic
fesskin	Hispanic
field nigger	Black
filipino	Asian
filipinos	Asian
fingernail rancher	Asian
fob	Asian
fuckmuslims	anti-islamic
ghanaian	Black
**ghetto**	Black
go back where	immigrant
gobackwhere	immigrant
golliwog	Black
gook	Asian
gookaniese	Asian
gookemon	Asian
gooky	Asian
groid	Black
guamanian	Asian
guatemalans	Hispanic
haitian	Black
haitians	Black
half breed	Multi-race
half cast	Multi-race
half-breed	Multi-race
half-cast	Multi-race
hatchet-packer	Native American
help refugees	refugees
hijab	anti-islamic
hijabs	anti-islamic
hindu	
hindus	
hispandex	Hispanic
hispanic	Hispanic
hispanics	Hispanic
house nigger	Black
illegal alien	immigrant
illegal aliens	immigrant
illegal immigrant	immigrant
illegal immigrants	immigrant
immigrant	immigrant
immigrants	immigrant
immigration	immigrant
**indian**	
indonesian	Asian
iranian	Middle Eastern
iraqi	Middle Eastern
iroquois indian	Native American
iroquois nation	Native American
iroquois tribe	Native American
**islam**	Middle Eastern
islamic	Middle Eastern
israeli	Middle Eastern
israelis	Middle Eastern
jamaican	Black
jamaicans	Black
**japanese**	Asian
**jewish**	
jews	
jig-abdul	anti-islamic
jigaboo	Black
jigga	Black
jiggabo	Black
jihad	Middle Eastern
jihads	Middle Eastern
jihadi	Middle Eastern
jihadis	Middle Eastern
jordanian	Black
kafeir	anti-islamic
karen people	Asian
kenyan	Black
knuckle-dragger	Black
**korean**	Asian
koreans	Asian
kuffar	anti-islamic
laotian	Asian
latin american	Hispanic
latina	Hispanic
latinas	Hispanic
**latino**	Hispanic
latinos	Hispanic
lebanese	Middle East
liberian	Black
little hiroshima	Asian
malayali	Asian
malaysian	Asian
mexcrement	Hispanic
**mexican**	Hispanic
mexicans	Hispanic
Mexican’t	Hispanic
mexico border	immigrant
mexicoborder	immigrant
mexicoon	Multi-race
mexihos	Hispanic
middle eastern	Middle Eastern
mongolian	Asian
mongolians	Asian
moroccan	Black
moroccans	Black
mozambican	Black
mud people	Black
mudshark	anti-islamic
**muslim**	Middle Eastern
muslimban	Middle Eastern
muslims	Middle Eastern
muzrat	anti-islamic
muzzie	Middle Eastern
native american	Native American
native americans	Native American
native hawaiian	Native Hawaiian
navajo	Native American
**negro**	Black
nepalese	Asian
nigerian	Black
nigerians	Black
**nigga**	Black
nigger	Black
niggers	black
nigglet	Black
nigglets	black
niglet	Black
noodle nigger	Asian
north korean	Asian
**oriental**	Asian
orientals	Asian
our country back	immigrant
ourcountryback	immigrant
pacific islander	Pacific Islander
paki	Middle eastern/south asian
pakistani	Middle Eastern
palestinian	Middle Eastern
panamanian	Hispanic
paraguayan	Hispanic
pashtun	Middle Eastern
pegida	anti-islamic
peruvian	Hispanic
pickaninny	Black
pisslam	anti-islamic
polynesian	Pacific Islander
porch monkey	Black
prairie nigger	Native American
pueblo indians	Native American
pueblo nation	Native American
pueblo tribe	Native American
puerto rican	Hispanic
puerto ricans	Hispanic
qtip head	anti-islamic
race traitor	Multi-race
raghead	anti-islamic
rag head	anti-islamic
rapefugee	anti-islamic
red nigger	Native American
refugee	refugees
refugees	refugees
resettlement	refugees
rice burner	Asian
rice nigger	Asian
rice rocket	Asian
rice-nigger	Asian
river nigger	Native American
rivernigger	Native American
rug pilot	Middle Eastern
rugpilot	anti-islamic
rug rider	Middle Eastern
rwandan people	Black
salvadoreans	Hispanic
samoan	Pacific Islander
sanctuary cities	immigrant
sanctuary city	immigrant
sanctuarycities	immigrant
sanctuarycity	immigrant
sand flea	anti-islamic
sand monkey	Middle Eastern
sand moolie	anti-islamic
sand nigger	Middle Eastern
sand rat	anti-islamic
sandflea	anti-islamic
sandmonkey	anti-islamic
sandmoolie	anti-islamic
sandnigger	anti-islamic
sandrat	anti-islamic
secure our border	immigrant
secureourborder	immigrant
shiptar	Middle Eastern
sioux indian	Native American
sioux nation	Native American
sioux tribe	Native American
slurpee nigger	anti-islamic
slurpeenigger	anti-islamic
somali	Black
somalian	Black
south african	Black
south american	Hispanic
south asian	Asian
sudanese	Black
sun goblin	Middle Eastern
syria	refugees
syrian	refugees
syrians	refugees
syrianrefugee	refugees
taco nigger	Hispanic
taiwanese	Asian
tanzanian	Black
tar baby	Black
tar-baby	Black
teepee creeper	Native American
tee-pee creeper	Native American
**thai**	Asian
thais	Asian
thin eyed	Asian
thin-eyed	Asian
tibetan	Asian
timber nigger	Native American
timbernigger	Native American
tomahawk chucker	Native American
tomahawk-chucker	Native American
tomahonky	Native American
towel head	anti-islamic
towelhead	anti-islamic
towel-head	Middle Eastern
undocumented	immigrant
unhcr	refugees
**vietnamese**	Asian
we welcome refugees	refugees
welcome refugee	refugees
welcomerefugee	refugees
wetback	Hispanic
whacky iraqi	Middle Eastern
whitegenocide	anti-islamic
wog	dark-skinned foreigner
zambian	Black
zimbabwean	Black
zipperhead	Asian
@artistsandfleas	exclude
negroni	exclude
new mexico border	exclude
deportes	exclude
deportiva	exclude
indiana	exclude
indianapolis	exclude
