# Supplementary material for: Pride, Love, and Twitter Rants: Combining Machine Learning and Qualitative Techniques to Understand What Our Tweets Reveal about Race in the US
Source: Int J Environ Res Public Health. 2019 May 18;16(10):1766. doi: 10.3390/ijerph16101766 (PMC6571562; doi:10.3390/ijerph16101766)
Supplement: Supplementary file 1 [file ijerph-16-01766-s001.pdf]

International Journal of
Environmental Research and Public Health

MDPI

Supplementary Materials

Pride, Love & Twitter Rants: combining machine learning and qualitative techniques to understand what our tweets reveal about race in the US.

Thu T. Nguyen, Shaniece Criss, Amani M. Allen, M. Maria Glymour, Lynn Phan, Ryan Trevino, Shrikha Dasari and Quynh C. Nguyen

Table S1. Race terms used in Twitter data Collection.

Items	Race
afghanistan	Middle Eastern
afghanistani	Middle Eastern
afghans	Middle Eastern
african american	Black
african americans	Black
african't	Black
africoon	Black
afro caribbean	Black
afro-caribbean	Black
aid refugees	refugees
alaska native	Alaskan Native
american indian	Native American
apache indian	Native American
apache nation	Native American
apache tribe	Native American
arab	Middle Eastern
arabs	Middle Eastern
arabic	Middle Eastern
arabush	Arab
**asian**	Asian
asians	Asian
asian indian	Asian
bahamian	Black
bahamians	Black
bamboo coon	Asian
ban islam	anti-islamic
ban muslim	anti-islamic
ban on mulsims	anti-islamic
bangalees	Asian
bangladeshi	Asian
banislam	anti-islamic
banjo lip	Black
banmuslim	anti-islamic
banonmulsims	anti-islamic
bantu	Black
beaner	Mexican
beaner shnitzel	Multi-race
beanershnitzel	Multi-race
bengalis	Asian
bhutanese	Asian
biscuit lip	Black
bix nood	Black

black boy	Black
black boys	Black
black female	Black
black girl	Black
black girls	Black
black male	Black
black men	Black
black women	Black
blacks	Black
bootlip	Black
borde jumper	Hispanic
border bandit	Hispanic
border control	immigrant
border fence	immigrant
border hopper	Hispanic
border nigger	Hispanic
border security	immigrant
border surveillance	immigrant
border wall	immigrant
bow bender	Native American
brazilians	Hispanic
buffalo jockey	Native American
build a wall	immigrant
buildawall	immigrant
bumper lip	Black
burmese	Asian
burnt cracker	Black
burundi	Black
bush-boogie	Black
bushnigger	Native American
cairo coon	Middle Eastern
cambodian	Asian
cambodians	Asian
camel cowboy	Middle Eastern
camel fucker	Middle Eastern
camel jacker	Middle Eastern
camelfucker	Middle Eastern
camel-fucker	Middle Eastern
cameljacker	Middle Eastern
camel-jacker	Middle Eastern
carpet pilot	Middle Eastern
carpetpilot	anti-islamic
carribean people	Black
caublasian	Multi-race
central american	Hispanic
chain dragger	Black
chamorro	Asian
cherokee indian	Native American
cherokee nation	Native American
cherokee tribe	Native American
cherry nigger	Native American
chexican	Multi-race
chicano	Hispanic
chicanos	Hispanic
chiegro	Asian
chinaman	Asian
**chinese**	Asian
ching-chong	Asian

chink	Asian
chinks	Asian
chippewa indian	Native American
chippewa nation	Native American
chippewa tribe	Native American
choctaw indian	Native American
choctaw nation	Native American
choctaw tribe	Native American
clit chopper	Middle Eastern
clit-chopper	Middle Eastern
clitless	Middle Eastern
clit-swiper	Middle Eastern
coconut nigger	Asian
colombian	Hispanic
columbians	Hispanic
congo lip	Black
congolese	Black
coonass	Black
coon-ass	Black
coontang	Black
costa rican	Hispanic
cracker jap	Asian
**cuban**	Hispanic
cubans	Hispanic
dampback	Hispanic
darkey	Black
darkie	Black
darky	Black
deport	immigrant
deportation	immigrant
deported	immigrant
deporting	immigrant
deports	immigrant
derka derka	anti-islamic
derkaderka	anti-islamic
diaper head	Middle Eastern
diaperhead	Middle Eastern
diaper-head	Middle Eastern
dog muncher	Asian
dog-muncher	Asian
**dominican**	Hispanic
dominicans	Hispanic
dothead	South Asians
dune coon	Middle Eastern
dune nigger	anti-islamic
dunecoon	anti-islamic
dunenigger	anti-islamic
durka durka	Middle Eastern
durka-durka	Middle Eastern
east asian	Asian
ecuadorian	Hispanic
egyptian	Black
egyptians	Black
end sanctuary	immigrant
ethiopian	Black
ethiopians	Black
fence fairy	Hispanic
fence hopper	Hispanic

fence-hopper	Hispanic
fesskin	Hispanic
field nigger	Black
filipino	Asian
filipinos	Asian
fingernail rancher	Asian
fob	Asian
fuckmuslims	anti-islamic
ghanaian	Black
**ghetto**	Black
go back where	immigrant
gobackwhere	immigrant
golliwog	Black
gook	Asian
gookaniese	Asian
gookemon	Asian
gooky	Asian
groid	Black
guamanian	Asian
guatemalans	Hispanic
haitian	Black
haitians	Black
half breed	Multi-race
half cast	Multi-race
half-breed	Multi-race
half-cast	Multi-race
hatchet-packer	Native American
help refugees	refugees
hijab	anti-islamic
hijabs	anti-islamic
hindu	
hindus	
hispandex	Hispanic
hispanic	Hispanic
hispanics	Hispanic
house nigger	Black
illegal alien	immigrant
illegal aliens	immigrant
illegal immigrant	immigrant
illegal immigrants	immigrant
immigrant	immigrant
immigrants	immigrant
immigration	immigrant
**indian**	
indonesian	Asian
iranian	Middle Eastern
iraqi	Middle Eastern
iroquois indian	Native American
iroquois nation	Native American
iroquois tribe	Native American
**islam**	Middle Eastern
islamic	Middle Eastern
israeli	Middle Eastern
israelis	Middle Eastern
jamaican	Black
jamaicans	Black
**japanese**	Asian
**jewish**	

jews	
jig-abdul	anti-islamic
jigaboo	Black
jigga	Black
jiggabo	Black
jihad	Middle Eastern
jihads	Middle Eastern
jihadi	Middle Eastern
jihadis	Middle Eastern
jordanian	Black
kafeir	anti-islamic
karen people	Asian
kenyan	Black
knuckle-dragger	Black
**korean**	Asian
koreans	Asian
kuffar	anti-islamic
laotian	Asian
latin american	Hispanic
latina	Hispanic
latinas	Hispanic
**latino**	Hispanic
latinos	Hispanic
lebanese	Middle East
liberian	Black
little hiroshima	Asian
malayali	Asian
malaysian	Asian
mexcrement	Hispanic
**mexican**	Hispanic
mexicans	Hispanic
mexican't	Hispanic
mexico border	immigrant
mexicoborder	immigrant
mexicoon	Multi-race
mexihos	Hispanic
middle eastern	Middle Eastern
mongolian	Asian
mongolians	Asian
moroccan	Black
moroccans	Black
mozambican	Black
mud people	Black
mudshark	anti-islamic
**muslim**	Middle Eastern
muslimban	Middle Eastern
muslims	Middle Eastern
muzrat	anti-islamic
muzzie	Middle Eastern
native american	Native American
native americans	Native American
native hawaiian	Native Hawaiian
navajo	Native American
**negro**	Black
nepalese	Asian
nigerian	Black
nigerians	Black
**nigga**	Black

nigger	Black
niggers	black
nigglet	Black
nigglets	black
niglet	Black
noodle nigger	Asian
north korean	Asian
**oriental**	Asian
orientals	Asian
our country back	immigrant
ourcountryback	immigrant
pacific islander	Pacific Islander
paki	Middle eastern/south asian
pakistani	Middle Eastern
palestinian	Middle Eastern
panamanian	Hispanic
paraguayan	Hispanic
pashtun	Middle Eastern
pegida	anti-islamic
peruvian	Hispanic
pickaninny	Black
pisslam	anti-islamic
polynesian	Pacific Islander
porch monkey	Black
prairie nigger	Native American
pueblo indians	Native American
pueblo nation	Native American
pueblo tribe	Native American
puerto rican	Hispanic
puerto ricans	Hispanic
qtip head	anti-islamic
race traitor	Multi-race
raghead	anti-islamic
rag head	anti-islamic
rapefugee	anti-islamic
red nigger	Native American
refugee	refugees
refugees	refugees
resettlement	refugees
rice burner	Asian
rice nigger	Asian
rice rocket	Asian
rice-nigger	Asian
river nigger	Native American
rivernigger	Native American
rug pilot	Middle Eastern
rugpilot	anti-islamic
rug rider	Middle Eastern
rwandan people	Black
salvadoreans	Hispanic
samoan	Pacific Islander
sanctuary cities	immigrant
sanctuary city	immigrant
sanctuarycities	immigrant
sanctuarycity	immigrant
sand flea	anti-islamic
sand monkey	Middle Eastern
sand moolie	anti-islamic

sand nigger	Middle Eastern
sand rat	anti-islamic
sandflea	anti-islamic
sandmonkey	anti-islamic
sandmoolie	anti-islamic
sandnigger	anti-islamic
sandrat	anti-islamic
secure our border	immigrant
secureourborder	immigrant
shiptar	Middle Eastern
sioux indian	Native American
sioux nation	Native American
sioux tribe	Native American
slurpee nigger	anti-islamic
slurpeenigger	anti-islamic
somali	Black
somalian	Black
south african	Black
south american	Hispanic
south asian	Asian
sudanese	Black
sun goblin	Middle Eastern
syria	refugees
syrian	refugees
syrians	refugees
syrianrefugee	refugees
taco nigger	Hispanic
taiwanese	Asian
tanzanian	Black
tar baby	Black
tar-baby	Black
teepee creeper	Native American
tee-pee creeper	Native American
**thai**	Asian
thais	Asian
thin eyed	Asian
thin-eyed	Asian
tibetan	Asian
timber nigger	Native American
timbernigger	Native American
tomahawk chucker	Native American
tomahawk-chucker	Native American
tomahonky	Native American
towel head	anti-islamic
towelhead	anti-islamic
towel-head	Middle Eastern
undocumented	immigrant
unhcr	refugees
**vietnamese**	Asian
we welcome refugees	refugees
welcome refugee	refugees
welcomerefugee	refugees
wetback	Hispanic
whacky iraqi	Middle Eastern
whitegenocide	anti-islamic
wog	dark-skinned foreigner
zambian	Black
zimbabwean	Black

zipperhead	Asian
@artistsandfleas	exclude
negroni	exclude
new mexico border	exclude
deportes	exclude
deportiva	exclude
indiana	exclude
indianapolis	exclude

Table S2. Count of tweets using race-related terms by state.

STATE	All Tweetes	Black	Middle Eastern	Hispanic	Asian
AL	15983	11405	669	1391	1990
AZ	21158	7514	1600	4723	3784
AR	5708	3680	179	968	704
CA	197514	69001	9823	41867	66897
CO	11880	4627	881	2406	3176
CT	11618	7269	375	1527	1727
DE	4823	3258	161	653	613
DC	18565	8227	2513	3184	3811
FL	98996	42420	7451	26184	19802
GA	56952	38855	2398	6575	7239
ID	1882	387	306	457	637
IL	39021	18233	2611	7788	8651
IN	13844	7823	1087	2435	2048
IA	4074	1607	245	882	1158
KS	5725	2558	383	1484	994
KY	9493	5426	395	1326	1563
LA	32354	27616	630	1626	2076
ME	1320	359	126	196	541
MD	33075	22474	2140	3261	4366
MA	20457	9093	1858	3600	4840
MI	33931	22473	2529	3104	4708
MN	9075	3509	686	1941	2493
MS	7573	6039	179	615	601
MO	12248	6323	720	1950	2682
MT	506	91	60	66	184
NE	3143	1598	190	556	649
NV	34063	6843	1096	9691	15800
NH	1460	468	74	392	396
NJ	37607	19262	2268	8259	6363
NM	3842	1204	173	899	500
NY	106644	34095	9138	23834	32527
NC	37413	21765	2471	4204	6078
ND	579	237	21	103	162
OH	40747	27147	2023	4386	5575
OK	9711	5345	675	1311	1515
OR	9163	1898	890	1443	4102
PA	41202	21895	3320	5149	9207
RI	3145	1701	109	516	685
SC	15416	10717	521	1668	2027
SD	847	226	135	113	158
TN	18288	10373	1018	2817	3256
TX	146947	92486	5944	26341	19320
UT	5413	1594	478	892	1769
VT	488	79	30	78	246
VA	32187	18199	2424	3446	6852
WA	18979	6223	1126	2796	7934
WV	2110	1063	139	311	510

WI	9907	4575	866	1800	2124
WY	459	68	34	145	149

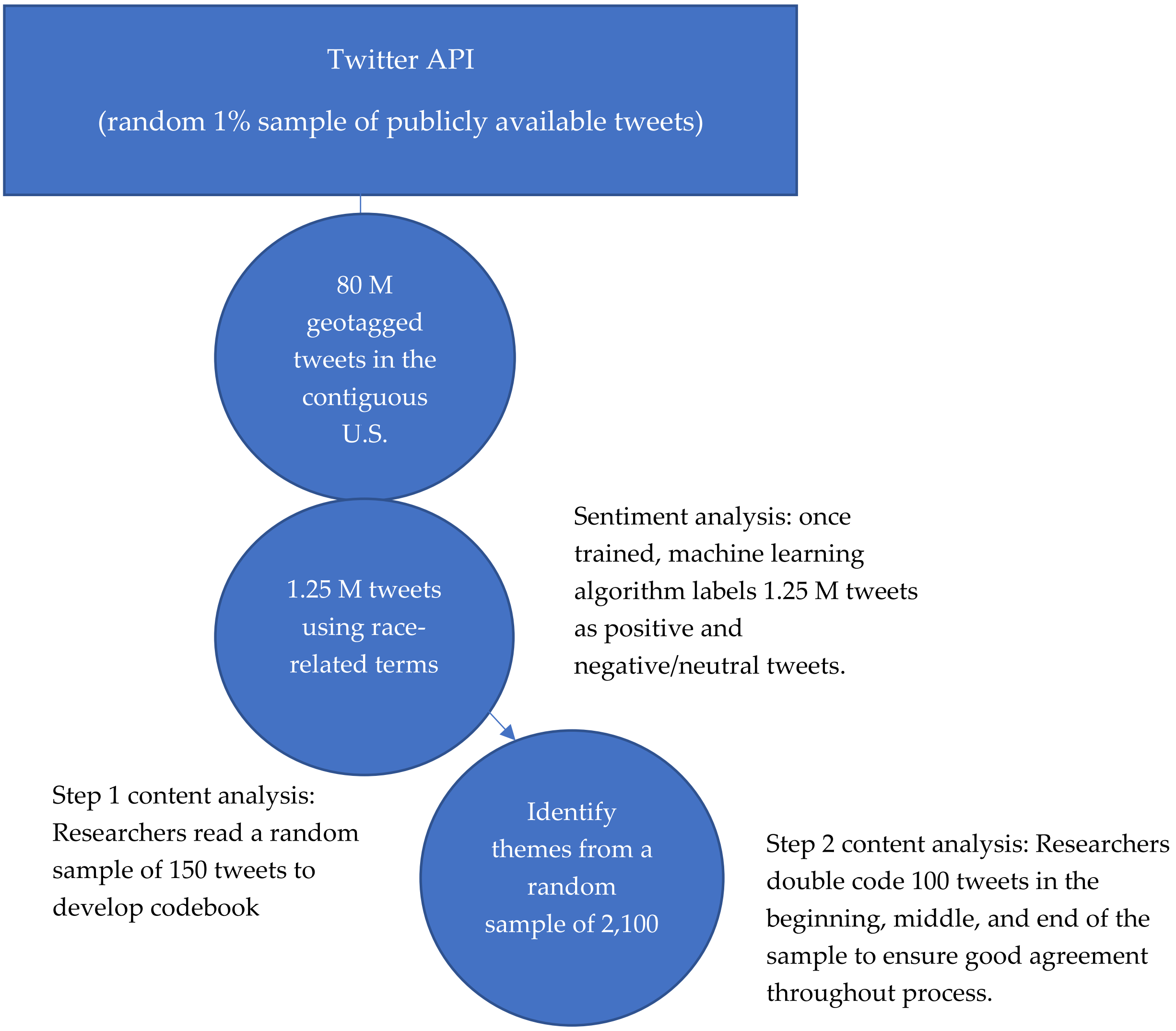

Figure S1. Analytic Sample and Analysis Flow Chart.

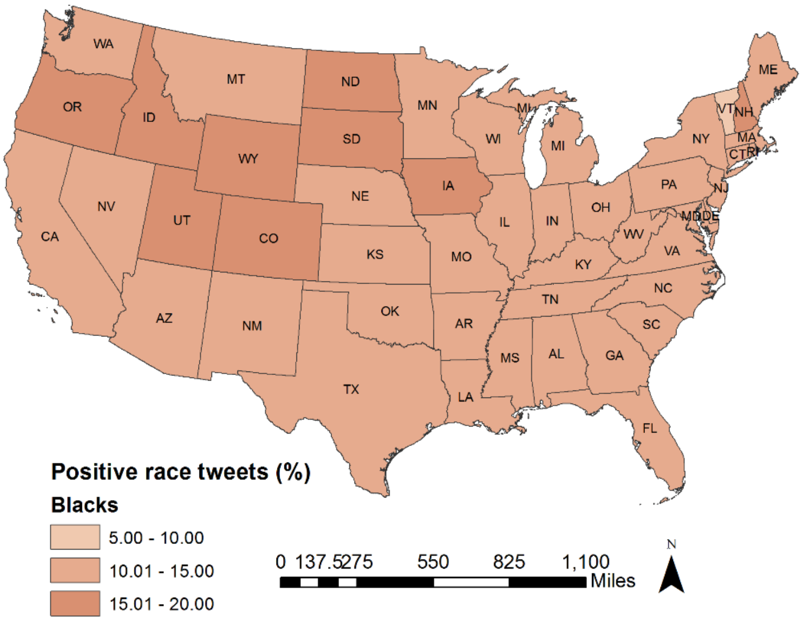

Figure S2. Geographic distribution of percent tweets using Black-related terms that are positive, collected April 2015-March 2016.

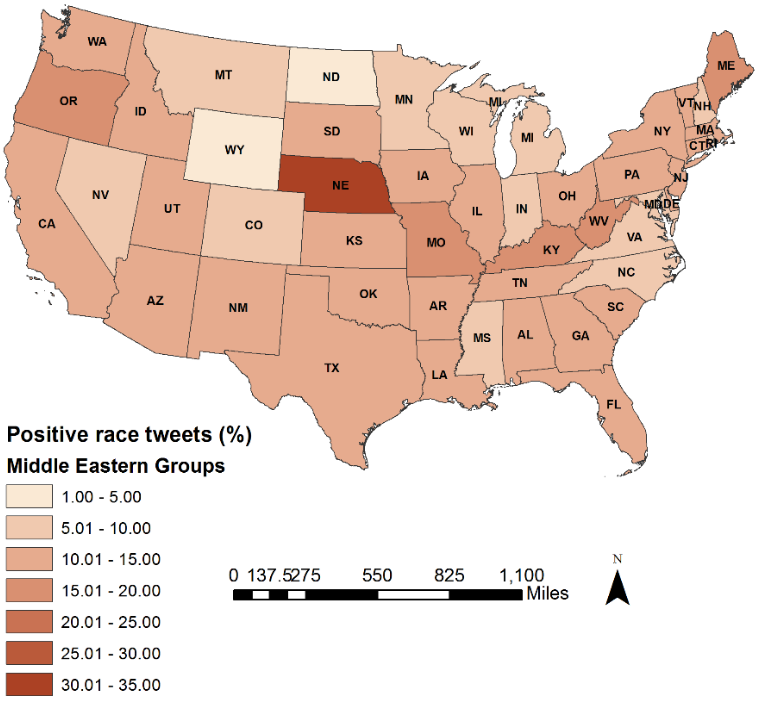

Figure S3. Geographic distribution of percent tweets using Middle Eastern-related terms that are positive, collected April 2015-March 2016.

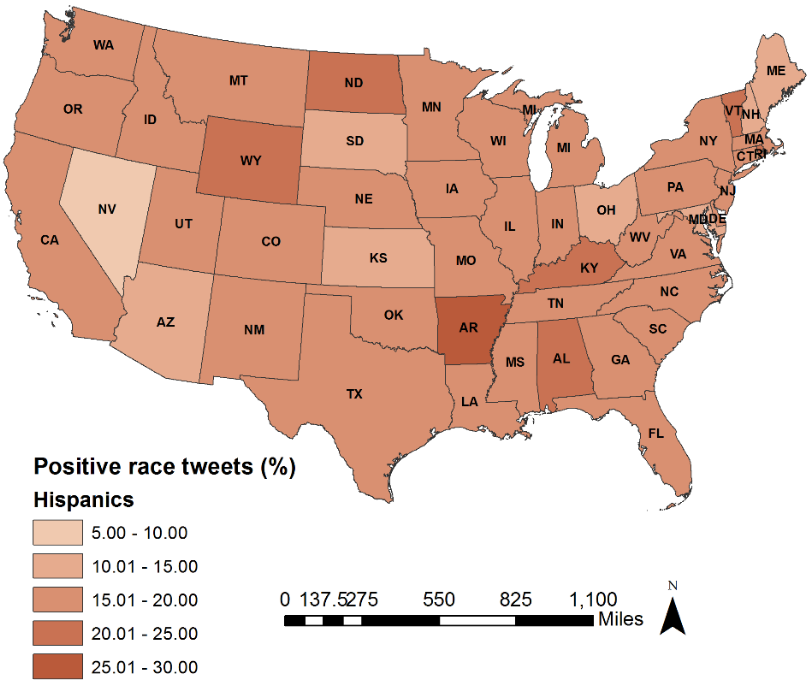

Figure S4. Geographic distribution of percent tweets using Hispanic-related terms that are positive, collected April 2015-March 2016.

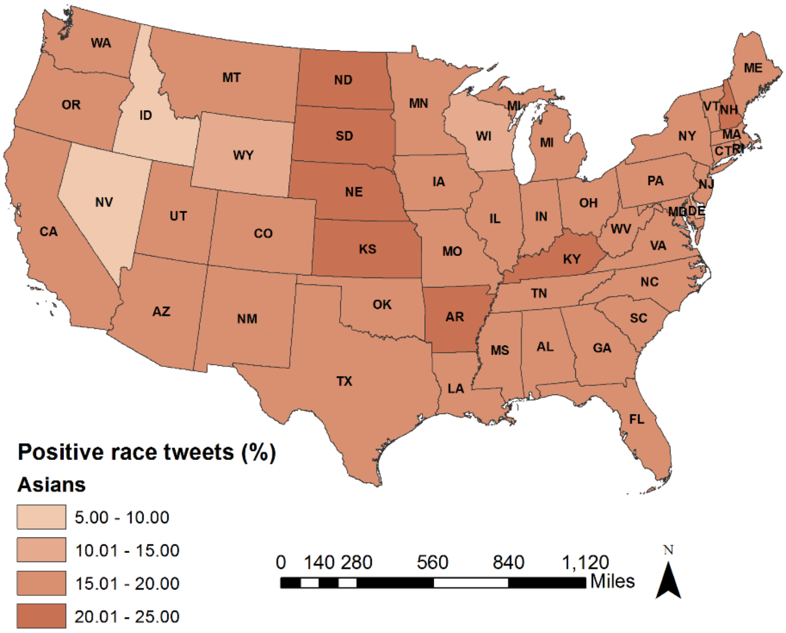

Figure S5. Geographic distribution of percent tweets using Asian-related terms that are positive, collected April 2015-March 2016.
